# Patient-Reported Experiences of Abortion Travel

**DOI:** 10.1001/jamanetworkopen.2026.27603

**Published:** 2026-08-06

**Authors:** Tuyet-Mai H. Hoang, Ainslee Wong, Hannah Baines, Radia DeLuna, Cache Monet Merriweather, Aaron Santoyo

**Affiliations:** 1 School of Social Work, University of Illinois Urbana-Champaign, Urbana; 2 College of Applied Health Sciences, University of Illinois Urbana-Champaign, Urbana; 3 Department of Educational Psychology, University of Illinois Urbana-Champaign, Urbana; 4 Department of Molecular and Cellular Biology, University of Illinois Urbana-Champaign, Urbana

## Abstract

**Importance:**

Illinois is an abortion hub serving a high volume of patients who travel from midwestern and southern states for abortion care. To sustain access to quality services and reduce health care barriers, research is needed to understand the experiences of abortion travelers.

**Objectives:**

To explore the experiences of out-of-state individuals who travel to central and southern Illinois to receive abortion care.

**Design, Setting, and Participants:**

This qualitative study used purposeful sampling to recruit 208 participants at 2 abortion clinics between November 2024 and August 2025. Participants completed the study at the time of recruitment.

**Exposure:**

Participants traveled for abortion care based on their state of residence.

**Main outcomes and measures:**

Using reflexive thematic analysis, both the experiences of out-of-state abortion travelers in central and southern Illinois and the need for reduction in access barriers for those traveling for abortion were examined.

**Results:**

In this qualitative study of 208 abortion seekers who successfully traveled to Illinois, 128 (61.54%) self-identified as Black, 50 (24.03%) non-Hispanic White, and 30 (14.42%) as women from additional minoritized racial and ethnic groups. Participants’ age ranged from 18 to 45 years. All lived in midwestern or southern states. Participants were interviewed in a private room at the clinic between November 2024 and August 2025. Participants discussed structural, logistical, and emotional challenges. Yet, they described abortion traveling as necessary because of the abortion ban and misinformation, which contributed to increased medical distrust. Medical distrust was already rooted in some participants’ previous experiences of receiving biased reproductive care, and for Black women, it worsened because of historical mistreatment and discrimination. Increased medical distrust likely drove people to travel for abortion care.

**Conclusions and relevance:**

These findings call for enhanced coordination of care with centralized trusted resources, equitable distribution of funding, mental health support, and attention to reduce biased care for Black women. The results also highlight the need to develop strategies to combat misinformation and increase advocacy efforts to reduce abortion stigma.

## Key Points

**Question:**

What are the experiences of people who successfully travel from other states to seek abortion care in central and southern Illinois?

**Findings:**

In this qualitative study, 208 abortion-seekers were interviewed and described facing structural, logistical, and emotional challenges. Participants described traveling for abortion as a need, not a choice.

**Meaning:**

These findings suggest a need for enhanced care coordination with trusted resources, emotional support, and consistent funding to sustain access and quality care in addition to increasing advocacy and developing strategies to combat misinformation.

## Teaser

This qualitative study investigates the experiences of out-of-state individuals who travel to central and southern Illinois to receive abortion care.

## Introduction

After the Supreme Court ruling in *Dobbs v Jackson Women’s Health Organization* in 2022,^1^ 13 states enacted immediate trigger bans on abortion.^2^ Among them, Missouri, Kentucky, and Tennessee border Illinois while Oklahoma, Texas, Arkansas, Louisiana, and Mississippi are located directly south of Illinois.^3^ Over the next 5 years, states surrounding Illinois continued to introduce provisions and enact policies to restrict access and instill fear around seeking abortion care.^2,3^As a long-standing protective state, Illinois had 21 500 abortions performed in the 12 months after the *Dobbs* decision, the highest in the country.^3,4^ Data from 2025 indicates it continues to be an essential hub for abortion seekers,^4^ and southern and central Illinois serve as the closest access point for patients from highly restrictive midwestern and southern states.^5,6^ To sustain Illinois as a haven for reproductive rights, more research is needed to understand the experiences of people who successfully travel to Illinois for care especially when sociopolitical context continues to shape emerging challenges.^7,8^ This study explores the impact of changing abortion provisions on the experiences of abortion seekers who travel to central and southern Illinois to design sustainable solutions and reduce health care burden. This study aims to examine the following research questions: (1) What are the experiences of out-of-state people who travel to seek abortion care in central and southern Illinois? (2) What are the needs of patients to reduce access barriers?

## Methods

This qualitative study obtained institutional review board approval from the University of Illinois Urbana Champaign, using purposeful sampling through 2 partnered clinics in central and southern Illinois. Clinics were chosen because they were the closest access points for people who travel for abortion care from highly restrictive states, such as Indiana, Tennessee, Missouri, and Mississippi. All participants completed the study at the time of recruitment. The interview questions were developed by the research team and community partners, and included topics on abortion access, barriers, and patients’ experiences with support systems (eg, family members, local community, and practical organizations). From November 2024 to August 2025, staff at each clinic distributed recruitment flyers to patients upon arrival so that they could review the materials while they waited for their appointment. They reiterated the voluntary nature of the study, the confidentiality guidelines, and the focus on patients who traveled to seek in-person abortion care. When participants expressed verbal interest, onsite research staff met with participants to discuss the purpose of the study and informed consent. Informed consent and self-reported demographic information were collected through a secure tablet using REDCap at this time. Next, semistructured interviews occurred in a private room with the research staff after participants’ clinical appointment. Interviews ranged from 20 to 40 minutes. Interviews were recorded using a secure device, and recordings were uploaded in Health Insurance Portability and Accountability Act–compliant online storage right after the interview and deleted from the device. Participants chose their own pseudonyms. At the end of the interview, participants received a resource list and contact information of the research team. This study followed Standards for Reporting Qualitative Research (SRQR) reporting guideline for qualitative studies to report the study’s procedure and findings.^9^

### Reflexivity

We recognized that our lived experiences and professional training shaped our interpretation and analysis of the data. Five of us are cisgender and 1 is transgender. Two are of Asian descent, 1 is Black, 1 is Hispanic White, 1 is of Latinx and North African descent, and 1 is White. Three of us are from social work, 1 from applied health science, 2 from psychology, and 1 from biology and medicine. Four of us used the lens of intersectionality and critical race frameworks to interpret the data, and 2 used trauma-informed perspectives.

### Data Analysis

We used a semistructured interview format and followed reflexive thematic analysis to allow for theoretical flexibility and account for the researchers’ lived experiences in understanding and interpreting the data.^10^ A latent thematic approach was used to examine the patterns underlying the diverse experiences of our participants through an iterative process of data familiarization, code generation, refinement, theme development, revision, and finalization.^11^ In the first 2 steps, all coders read the transcripts and worked in a group of 2 to generate initial codes from 5 interviews. All coders met and refined the codes further. Next, double coding was completed by T.M.H.H. and R.D. for another 15 interviews, which reached sufficient data saturation to finalize the codebook.^12^ Data were then evenly divided into 3 sections with 2 coders per section. All coders worked independently and made reflective notes; the team met in total 17 times for 2 to 3 hours each time to examine latent themes, identify exemplifying quotes, and review similarities and discrepancies. In the event that coding discrepancies were not resolved in individual meetings between the 2 coders, discrepancies were addressed through discussions with the entire coding team, where each of the 6 coders reviewed and discussed their interpretation of the data until a consensus was achieved. We provided percentages of people who stated they did not have follow-up care and those people who chose not to disclose their abortion. These questions were asked in the interview guide (eAppendices 1 and 2 and eMethods in Supplement 1). We coded for patients’ verbatim answers and quantified them by calculating the percentages out of total sample. Data were transcribed verbatim by A.S. and T.M.H.H., and the research team used MAXQDA version 2024 (VERBI Software) to conduct data analysis.

## Results

This study included 208 abortion seekers from midwestern and southern states with age ranged from 18 to 45 years. Participants self-identified as American Indian or Alaska Native (1 [0.48%]), Asian or Pacific Islander (5 [2.40%]), biracial or multiracial (12 [5.77%]), Black (128 [61.54%]), Hispanic White (11 [5.29%]), Middle Eastern or North African (1 [0.48%]), and non-Hispanic White (50 [23.79%]). Missing data in demographics were home state (5 participants), community type (4 participants), gender (4 participants), race (4 participants), and age (3 participants). A detailed summary of participants’ demographic information can be found in Table 1. In the Global Theme section, we described 3 global themes with subthemes and illustrative quotes. Table 2 includes additional illustrative quotes.

**Table 1.** Participant Demographics

Characteristics	Participants, No. (%)
Total No. of participants	208
State	
Indiana	74 (36.10)
Tennessee	49 (23.90)
Mississippi	20 (9.76)
Kentucky	15 (7.32)
Arkansas	10 (4.88)
Alabama	7 (3.41)
Georgia	6 (2.93)
Ohio	6 (2.93)
Missouri	6 (2.93)
Louisiana	5 (2.44)
Iowa	3 (1.46)
Texas	2 (0.98)
Florida	1 (0.49)
Michigan	1 (0.49)
Race	
American Indian or Alaska Native	1 (0.48)
Asian or Pacific Islander	5 (2.40)
Black	128 (61.54)
Biracial or multiracial	12 (5.77)
Hispanic White	11 (5.29)
Middle Eastern or North African	1 (0.48)
Non-Hispanic White	50 (23.79)
Age, y	
18-25	88 (42.50)
26-35	98 (47.34)
36-45	20 (9.66)
Community	
Large city	71 (34.47)
Small city	52 (25.24)
Suburb near a large city	46 (22.33)
Rural area	37 (17.96)

**Table 2.** Overarching Themes and Subthemes With Illustrative Quotes

Subtheme	Illustrative quote examples
**Theme 1: structural and logistical barriers**	
Abortion ban and criminalization	I was too scared, too, because I didn’t. I thought I was going to get in legal trouble with the state that I live in. And that’s where I was like, I don’t know what to do. I was even scared to call. And it’s a lot of other service there that [used to be] for free. They were helping us like birth control and health. That is not anymore. They banned them. So now almost everything you got to pay and for some people, they don’t have that much money. It’s a little bit more hard to get in that service because they banned them. It’s no more.
Legal changes and misinformation	It was very difficult because even not even just my state, like a lot of the southern states do not provide abortions. Like if I could have just went to like Florida, that’s like 3 hours away from me. They provide you an abortion, but only up to 6 weeks of pregnancy. And most of the time you don’t find out until you’re about 6 weeks pregnant. And then a lot of places, like what they don’t tell you is a lot of states you have to have 2 appointments before the 6 weeks. The crisis clinics or whatever they are, they don’t actually provide abortion care but rather, you know, they kind of trick people into coming there.
Logistical challenges and inconsistent funding information and availability	So everybody was like, oh, [our clinic does] medication [abortion] only. Or they would be like, oh, we can only do like a 7am appointment. And like for that we have to leave at like 5….It was…really frustrating to get the times lined up and like every time we’d just like be doing the math. Okay. Two hours to get there. This is how long the procedure is, you know. But what time change? All the funding places I would get in touch with, they would take days, weeks to respond to me. And like, sometimes it could only cover, like, it would tell me they could only cover like 300 [dollars] or, you know, maybe 400. But at that time, the cost was about 2000.
**Theme 2: increased medical distrust and the need to travel for care**	
Medical distrust	[Clinicians in my state] are like purely white. Like white white to the point to where they don’t know that Black people have feelings. Like they don’t know when we bleed that that hurt it. They probably think we bled and then blood just coming out and there’s nothing there. ● I have to be really careful with everything I say to any provider. So it’s felt like you’ve had to be kind of cautious of people just because of their opinions. Like I’ve heard that…It’s kind of frustrating because you’re supposed to be your doctor, so you’re supposed to like be able to tell them things. And I feel like I’m not comfortable telling those people.
The need to travel for abortion care	I feel like follow-up care…I definitely also feel equally comfortable just traveling back up here instead of, you know, because like if it doesn’t work 100% or if any complications whatsoever, I would feel more comfortable either coming back to where I was here. If I choose to make the drive again, so more like traveling to get follow-up care rather than it being there. Because last time I did wind up going to the ER because the cramping and stuff got severe and one of the nurses reported to the police, so I had to deal with that and it was just awful.
**Theme 3: internalized social stigmas and psychological burden**	
Shame and guilt behind nondisclosure	I just didn’t tell them. I don’t want to say it’s like a shameful secret, but, like, it feels more Like a keep it secret to keep us safe. Safe, like, because, like, it is. I don’t know. It wasn’t an easy decision for us and, like, I will think about it occasionally for the rest of my life. But I didn’t need my family and my community shoving down my throat that I was a baby killer and I’m all these other terrible things. I’m like, I know it’s the right thing. Absolutely. I cry about it and I’m hurt. Like, I have not been able to talk myself out of it and I have not been able to, like. Like, I know it’s the right thing for me and my family. So I just haven’t told anybody. My mom was the main person to tell me, you shouldn’t [have an abortion]. You should just accept your death. And that kind of hit hard because you’re my mom. You gave me life.
Internalized responsibilities and stigmas	And I remember crying about it for days and all those emotions, but it kind of woke me up. And it was like. A lot of people look at women, unfortunately, because they have that old school mindset that we are just human incubators, that we were put on this earth to make babies, be good housewives and do what we have to do. And that was it. And it kind of took me aback and woke me up like, whoa, that’s not what I am. I’m a human being. I have rights. But, yeah, [my partner]. He gets very. He gets very emotional about this stuff. So, like, when we got here, he was already just bawling out. I was like, you cannot do this while going in here. Please don’t. That’s why, like, I’m trying to keep, you know, a happy vibe. And I cry a lot because I don’t feel proud of what I’m doing right now. I cry a lot, but I think that’s the best that I can do right now…Also, I was, I was taking pills, so I don’t know what happened. Like, because it’s my fault. I know I should be more careful. But it was maybe accident. I was taking pills, but the doctor says not 100%, so exactly.

Abbreviation: ER, emergency room.

### Global Theme 1: Structural and Logistical Barriers

#### Abortion Ban and Criminalization

All participants said that abortion law was the biggest barrier to care, which contributed to their need to travel and increased fear of criminalization and distress. Many participants expressed a fear of being reported when they considered going to a local clinic or seeking resources from practitioners in their home states. One participant described her experience:

Like, if I was to get something go wrong with the abortion and I need to seek medical attention, how do I tell them that? You know what I mean? Like, without lying, like, because I don’t like to lie. But I mean, in Texas, that’s your only choice. Don’t want to be on the bad ends with law enforcement or if you don’t just want an enemy right there, you know, the doctors. I mean, people are cruel, especially when they feel like, you know, you’re going against their beliefs.

Others were afraid to type into a search engine because of data privacy concerns. One participant explained: “I was, like, kind of paranoid, Googling stuff…Just because I went and got ultrasounds in Arkansas, and I was like, I don’t know how illegal this is. And I’m not gonna Google it…I was, like, getting scared to even type the word [abortion].”

There was also confusion about telehealth and fear of receiving it, which could result in arrest for illegal drug possession. One participant spoke about telehealth: “Telehealth. Yes. Because it’s [abortion is] illegal in Indiana. I think the thing is, like, I can’t send it to Indiana because it’s like, like taking drugs over the border…Like, you can get arrested for having that in your car, even though you bought it from somewhere else.”

#### Legal Changes and Misinformation

Furthermore, abortion provisions were changing quickly at times, which created confusion and misinformation about services and risks among participants. One person explained: “At one point, [in] Georgia, this is how it went…You could have an abortion up to 24 weeks in Georgia, then it went to 6 weeks, then literally for 3 weeks, 3 days, and went back to 20 weeks. So, I feel as though you’re playing with people’s lives.”

Information access challenges contributed to delayed access. One participant described looking at online information and feeling overwhelmed, saying: “And everything is just so sketchy now, you can go to the internet, but it’s so much. I can’t believe everything on the internet. So, like you don’t know who or what to trust when you’re trying to find care or even considering who to tell.”

#### Logistic Challenges and Inconsistent Funding Information and Availability

Logistical barriers have always been a problem for abortion travelers. Logistical challenges included cost of the procedure, distance, finding a ride, road safety, car condition, clinic scheduling, and weather risks. One participant referenced several logistical challenges:

This is 2 hours and 8 minutes [travel time from my home]. Paying for gas. You know, if you like, if you had to find like childcare…I had to get an oil change…Literally Friday, I had to get my oil changed. I had to get an appointment for that. It was just so hectic the last 48 hours, honestly.

Another associated challenge was the inconsistency in funding information and availability, which intensified logistical planning. Information about funding appeared to be varied and dependent on what the clinic staff offered during time of contact, as well as the time of month and year when funding fluctuated. Some patients were offered a reduction in service cost, while others were not. Some patients were connected to abortion funds right away, while others mentioned they did not even know about abortion funding support. One participant mentioned that she had never received any information about funding for people who could not afford care: “I booked my appointment. Like I said, I rescheduled 3 times. Not once has anybody said, there’s also financial help for you if you can’t afford it or other options.”

### Global Theme 2: Increased Medical Distrust and the Need to Travel for Care

#### Medical Distrust

Participants reported increased medical distrust stemming from biased care that they personally experienced. One participant explained her experiences in seeking reproductive care at home: “I wouldn’t even be going through this if I wouldn’t have lost my Medicaid and if my OBGYN would just let me get my tubes tied. But since I have 2 girls. We can’t do that. You’re not old enough. Interesting. I’m 26 years old. I think I can make my own decisions.”

Medical distrust was also rooted in historical mistreatment and discrimination among Black women. One Black woman described her previous experience with health care practitioners at home:

What do you think about this narrative of like Black women dying during maternal issues? You know, it is, that is very much true because even when I was in the hospital, I’m glad I had a Black nurse because she’s the one who noticed my epidural hadn’t kicked in and nobody had been in to check on me. And so when she came in and she saw that I was still in pain, she went to get the anesthesiologist, and they found out they put it in the wrong spot.

#### Traveling for Abortion Care Became Necessary

About 50% (102) of participants did not have a plan for follow-up care at home. These patients indicated that they would travel back for follow-up if something was to happen. In addition, Black participants stated that they had no choice but to travel for care because of medical distrust and mistreatment they faced in the health care system. Participants had chosen the clinic as carefully as they could, and many people reported seeking referrals from someone they knew or trusted because of medical distrust. One Black participant discussed her need to travel to seek quality, respectful, and safe care: “I have access to healthcare, but not the open and honest safe health care that would be here. If this [abortion] was not to be completed and I needed to come back for the hospital, like, I would want to come back here. At least Illinois. Like, somewhere where it feels safer.”

### Global Theme 3: Internalized Social Stigmas and Psychological Burden

#### Shame and Guilt Behind Nondisclosure

All participants reported some level of family and/or partner support, however, 133 (64%) also indicated that they were keeping their abortion a secret from all family members. They described feelings of shame and guilt because of abortion stigma and social biases. One participant said: “Abortion is frown[ed] upon a lot in Mississippi. So I just didn’t say anything [to anyone]. My mom doesn’t even know now…I don’t want to hurt my mom. No, I can’t hurt her…I feel like there’ll be a major disappointment. I just can’t.”

#### Internalized Responsibilities and Stigmas

Participants also discussed their own responsibility and emotional burden for their reproductive outcomes. They expressed feeling responsible for the emotional regulation of family members, which contributed to the masking of their own emotions and distress. Some reported needing to be strong to protect their families and to overcome challenges. One participant said that in her friend group she’s “the strong one” and that her friends wouldn’t know even if she was “going through a mental breakdown…because I always kind of make it look seem like I got it together.” One participant contextualized abortion stigma for Black women: “I think Black women get a lot of the [stigmatizing] narrative, not even just with abortion. It’s like they say, we are the ones who [get] the most welfare and all of that and not even realize it’s harder for us to get it because we’re Black.”

### Recommendation Based on Patients’ Experiences and Barriers

Table 3 provides a full list of specific recommendations that correspond to patients’ experiences and global themes. All participants indicated they wanted abortion restrictions to be removed. They asked for an enhanced process in coordination of care with access to trusted information and resources on abortion services and risks. One participant asked:

**Table 3.** Recommendations Based on Patients’ Experiences and Barriers

Global themes	Recommendation
Theme 1: structural and logistical barriers	Clinics should try to coordinate with abortion funds and practical support organizations to strategically distribute more funding toward the time of the year where there is higher volume of patient. Clinic staff should have a standardized conversation about funding sources and distribution of funding with patients to increase transparency. Clinics should introduce basic follow-up care such as simple return phone calls to check on patients. Clinics should provide information about trustworthy telehealth abortion follow-up care accessible in patients’ state. Researchers should work with clinics and stakeholders to centralize trusted information to address confusion and misinformation. Policymakers should continue to collect data or work with researchers to advocate for legal changes or strategies to combat misinformation.
Theme 2: increased medical distrust and the need to travel for care	Clinics continue to provide quality and safe care as well as build support network with local community advocacy groups. Local community advocacy groups, abortion funds and practical support organizations work together to reduce the burden for coordination of care for in-person abortion facilities. Community advocacy groups focus on conducting outreach events to increase awareness and reduce abortion stigma.
Theme 3: internalized social stigma and psychological burden	Clinics should train staff to have transparent and empathic communication. Clinics should consider hiring social workers or patient counselors to provide some emotional support. Clinics should provide local resources for mental health support or provide secure and safe hotline information.

How can you discuss [abortion] with a doctor, you know, someone in Mississippi when it’s illegal? What do you say? Like do y’all know anywhere I can go in, you know, like having access to something, I guess to the resources from a trustworthy place would have been helpful.

Participants requested more transparency and consistent funding support. They emphasized the need to access safe and quality care. Additionally, participants also asked for more mental health support and empathic care without fear of prosecution and normalization of the experience of having an abortion: “I think…that way people don’t feel, you know, so alone. Like, you know, people…should be able to talk freely and openly and, you know, not be scared of, like, getting in trouble or being judged.”

## Discussion

The findings of this qualitative study confirmed existing research and that the most salient concerns post-*Dobbs* were that abortion restrictions and rapid legal changes that could result in an increased fear of criminalization and logistical barriers.^13,14^ Although cost, long traveling distances, and logistical challenges have been consistently reported,^15,16,17,18^ changes in sociopolitical context contribute to intensify social stigmas and misinformation.^19,20^ These cumulative evolving challenges further delay access to care, worsen existing medical distrust, and drive the need to travel for abortion care, especially among low resource groups. There is a need to improve care coordination by increasing patient access to trusted resources and follow-up care. Improved coordination can help sustain quality in-person abortion access in critical locations like central and southern Illinois,^21^ which remains needed even with telehealth as legal restrictions persist across all domains of abortion access.

Anti-abortion laws also increase significant burden on the most vulnerable groups. Overall, 71% of our sample consisted of women of color, and their experiences reflected multiple systems of inequities. These women reported a high level of the vulnerability due to experiences with gendered racism and socioeconomic disadvantage.^22,23,24,25^ Access to timely funding was critical for these women, but based on the findings, this process varied depending on what was offered during time of contact, as well as funding fluctuations based on the time of month and year. We urge abortion funds, practical support organizations and clinics to track patient volume and distribute funding toward the time of the months and year where there is higher volume of abortion seekers to reduce disparities.^4,26,27^

Furthermore, our findings indicated that emotional toll not only affected pregnancy, childbearing, or abortion, it also encompassed internalized racism, sexism, and classism.^25^ These structural factors also shaped beliefs about abortion, gender-based, race-related, and social stigmas among participants and their communities, increasing abortion seekers’ tendency toward secrecy, isolation, distrust, and distress.^28,29^ To reduce emotional stress, staff should be trained to provide transparent and empathic communication or clinics should consider having an on-site social worker or patient counselor. At a larger picture, there is a need to address biased narratives about reproductive health through advocacy efforts as well as strategies to combat misinformation that harm people’s health and mental health in accessing care.^20,30^

### Limitations

This study has some limitations. For example, findings were gathered at just 2 clinics, both of which were relocated from other states, Tennessee and Indiana, and as a result they attracted abortion-seeking travelers from those states disproportionately. Participants might also experience recall bias.

## Conclusions

In this qualitative study of people who traveled to Illinois for abortion care, our findings extended previous research by documenting the cascading associations of how abortion bans and the changing legal landscape intensified misinformation and fear of criminalization.^13,20^ These structural factors exacerbated logistical barriers and the emotional toll for out-of-state patients who traveled for care. Abortion travelers also reported increased levels of medical distrust. The results demonstrate a need to develop an enhanced coordination of care with trusted information to sustain quality in-person abortion access, which is necessary. The findings also call attention to more research and advocacy efforts to combat misinformation and social stigmas.

## Notes

Supplement 1. eMethods 1. Individual interview introduction eMethods 2. Interview protocol eAppendix. Resource list

Supplement 2. Data Sharing Statement
