# Supplementary material for: Patient-Reported Experiences of Abortion Travel
Source: JAMA Netw Open. 2026 Aug 6;9(8):e2627603. doi: 10.1001/jamanetworkopen.2026.27603 (PMC13448791; doi:10.1001/jamanetworkopen.2026.27603)
Supplement: Supplementary file 1 [file jamanetwopen-e2627603-s001.pdf]

Supplemental Online Content

Hoang TH, Wong A, Baines H, DeLuna R, Merriweather CM, Santoyo A. Patient reported experiences of abortion travel. *JAMA Netw Open.* 2026;9(8):e2627603. doi:10.1001/jamanetworkopen.2026.27603

eMethods 1. Individual interview introduction

eMethods 2. Interview protocol

eAppendix. Resource list

This supplemental material has been provided by the authors to give readers additional information about their work.

© 2026 Hoang TH et al. *JAMA Network Open*.

eMethods 1. INDIVIDUAL INTERVIEW INTRODUCTION

Introduction. Thank you so much for agreeing to participate in this interview focused on describing the experiences of traveling for abortion care in today's sociopolitical context. My name is [moderator name] and I am a researcher in the School of Social Work at the University of Illinois at Urbana. In just a minute, we can start the interview, but first I'd like to go over a few things with you. Your input and insights will be very useful to us as we analyze the experiences of people who travel for abortion care post Roe v. Wade to inform the creation and improvement of resources available to patients like you. Just so you know what to expect, we thought we would first outline the interview process, go over the consent form, and answer any questions you may have before we actually begin with the interview or conversation.

Purpose & Procedure
I want to begin by thanking you for taking time out of your schedule to talk with me today. What we are doing today is an individual interview to learn about your experiences, thoughts, and perceptions. Hearing from you is important and there are no right or wrong answers. All of your contributions to the discussion are valued. The interview will last about 40-60 minutes.

Do you have any questions for me? And is there any part that you want me to repeat?

Informed consent. Before we begin, we would like to describe your rights as a participant in this study in more detail. We'd like to quickly go over the main points in the consent form.

- Participation is completely voluntary – meaning you have the right to not answer questions in the interview if you do not want or you can stop the interview at any time if you choose.
- The interview will be audio-recorded – to make sure that we are able to accurately capture your perspectives, we will digitally record the interview. The digital recording will not have your name on it anywhere. After the interview is completed, we will download the interview and place it into a password protected file. The digital files will be destroyed five years after this project is completed.
- Participation is confidential – several safeguards will be taken to protect your identity. We will not talk about the details of this interview to others. We will, however, have a small group of trained graduate students and research professionals who will be working on various parts of the project. This will include activities such as transcribing the interviews and helping identify themes in the interviews. We will not report any information that could potentially be damaging to you. We cannot guarantee that other participants in the focus group will not disclose information outside of this room. We ask that you respect others' confidentiality and not discuss the content of others' comments outside of this room. Finally, we will ask that you choose a pseudonym, or fake name, to use during your participation in our focus group today.

© 2026 Hoang TH et al. *JAMA Network Open*.

- Want more information – if you would like to learn more about your rights as a participant in research feel free to call the IRB office at UIUC. The numbers are provided on the consent form.
- Keep a copy for your records – please read over the consent form and sign; here is a copy of the form for your records.
- Resource list – here is a resource list in case you would like to further explore the issues we discuss in these interviews
- Token of appreciation – as a token of appreciation for your participation in the study, you will receive $100 (US).
- **Questions**. We are about ready to begin the interview. Do you have any questions before we start, and do you give your consent to proceed?

eMethods 2. INTERVIEW PROTOCOL

Introduction

Today, we're going to be talking about your experiences traveling for abortion care. We are interested in your experiences, as your feedback will inform us of our recommendations for improving resources available for traveling patients.

INDIVIDUAL INTERVIEW QUESTIONS

1. How has the ban of abortion right affected your experience with getting abortion and family planning services?
2. What difficulties did you face when trying to get abortion care?
3. Were you worried about the cost or travel for getting care?
4. Were you concerned about how this would affect your family or how your community would react?
    a. Can you expand on your reason to share or not to share?
5. Were you able to take time off work if needed?
6. Did you have access to follow-up care if you needed it?
    a. Can you expand more on that?
7. Do you feel like you have a support system at home or in your community?
8. How was your experience finding a provider or scheduling an appointment?
9. What support or resources do you wish were available to help you feel better?
10. What changes do you think should happen right away?
11. Have you ever felt unsafe during this process?
    1. What do you need to feel safer?
12. We've covered a lot today. Do you have any questions? (ANSWER QUESTIONS). Is there anything else you'd like to add that we didn't talk about today?

© 2026 Hoang TH et al. *JAMA Network Open*.

Thank you all for sharing your experiences. I have learned a lot from you and the feedback you provided on your experiences will help shape recommendations for improving the experiences of support agencies.

Common Probes We May Use:

- Tell me more about that.
- That's really interesting, can you expand more on that?

eAppendix. **RESOURCE LIST**

Thank you for your participation in this study. Information collected from this project will help us gain a better understanding of the experiences of patients who travel for abortion care.
Some of the issues addressed in this focus group discussion may be difficult to deal with or may bring up additional questions. If you would like to talk to someone more about these topics, here are some places you can contact:

Mental Health Resources

- **Community Elements Crisis Line** **(217) 359-4141**
- **Crisis Text Line** **Text "HOME" to 741741**
- **National Council for Mental Wellbeing**
  - https://www.mentalhealthfirstaid.org/mental-health-resources/

Educational Resources

Here are some educational resources that provide additional information on some of the topics covered in this research project:

Janiak, E., Freeman, S., Maurer, R., Berkman, L. F., Goldberg, A. B., & Bartz, D. (2018). Relationship of job role and clinic type to perceived stigma and occupational stress among abortion workers. Contraception, 98(6), 517–521. https://doi.org/10.1016/j.contraception.2018.07.003

O'Donnell, J., Weitz, T. A., & Freedman, L. R. (2011). Resistance and vulnerability to stigmatization in abortion work. Social science & medicine (1982), 73(9), 1357–1364. https://doi.org/10.1016/j.socscimed.2011.08.019

Web Resources

- **National Abortion Federation -** https://prochoice.org/naf-membership/
  - Membership, networking opportunities for abortion providers
- **Society of Family Planning** - https://societyfp.org/community/membership/
  - Networking, mentorship, community engagement opportunities for abortion providers
- **American Medical Association Professional Well-Being Resources**

© 2026 Hoang TH et al. *JAMA Network Open*.

- Physician Well-Being Educational Module - https://edhub.ama-assn.org/steps-forward/module/2702556
- Stress First Aid for Health Care Professionals - https://edhub.ama-assn.org/steps-forward/module/2779767?resultClick=1&bypassSolrId=J_2779767

Abortion Support Resources

- general abortion support
  - https://www.all-options.org/
- post-abortion circles and other resources for people especially obtaining abortion in Illinois
  - https://www.chicagoabortionfund.org/
- faith based abortion support
  - https://www.faithaloud.org/
  - https://rcrc.org/
