# Supplementary material for: Patient-Reported Experiences of Abortion Travel
Source: JAMA Netw Open. 2026 Aug 6;9(8):e2627603. doi: 10.1001/jamanetworkopen.2026.27603 (PMC13448791; doi:10.1001/jamanetworkopen.2026.27603)
Supplement: Supplementary file 2 [file jamanetwopen-e2627603-s002.pdf]

Data Sharing Statement

Hoang. The Lived Experiences of Abortion Travel. *JAMA Netw Open*. Published August 06, 2026. doi:10.1001/jamanetworkopen.2026.27603

Data
Data available: Yes
Data types: Deidentified participant data
How to access data: Data can be requested to thhoang3@illinois.edu
When available: With publication

Supporting Documents
Document types: None

Additional Information
Who can access the data: Researchers whose proposed use of the data has been approved.
Types of analyses: qualitative analysis.
Mechanisms of data availability: Data will be made available with a signed data access agreement and with investigator support.
